# Supplementary material for: Circadian control of hepatitis B virus replication
Source: Nat Commun. 2021 Mar 12;12:1658. doi: 10.1038/s41467-021-21821-0 (PMC7955118; doi:10.1038/s41467-021-21821-0)
Supplement: Supplementary file 1 — Supplementary Information [file 41467_2021_21821_MOESM1_ESM.pdf]

Supplementary Information

Circadian control of hepatitis B virus replication

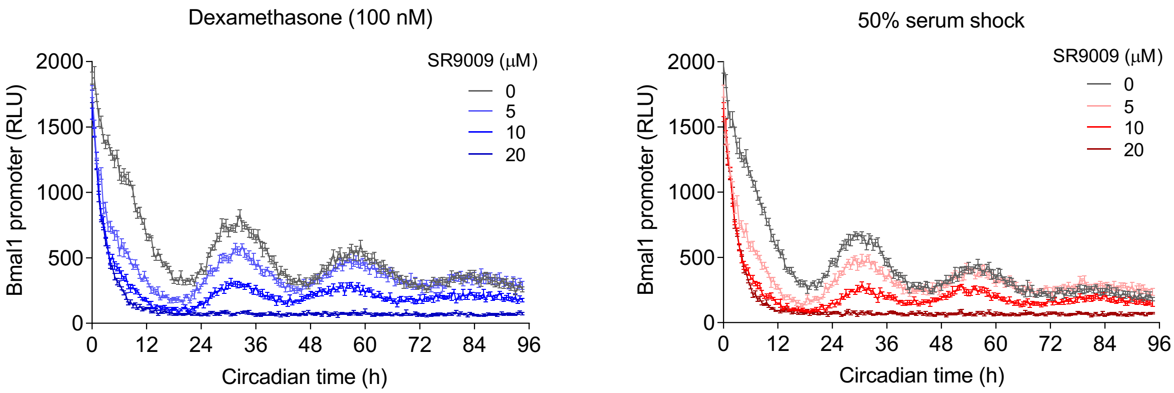

Supplementary Fig.1: Synchronization of HepaRG cells.
HepaRG cells stably expressing Bmal1 promoter luciferase reporter were synchronized with dexamethasone at 100 nM or 50% FBS for 2h. After removal of dexamethasone or FBS, cells were cultured in media containing live substrate in the presence of an increasing dose of REV-ERB agonist SR9009. Live luciferase signal was monitored at 30 min intervals for 96h. Data are expressed as the mean relative light units (RLU) ± S.E.M., n = 6. Source data are provided as a Source Data file.

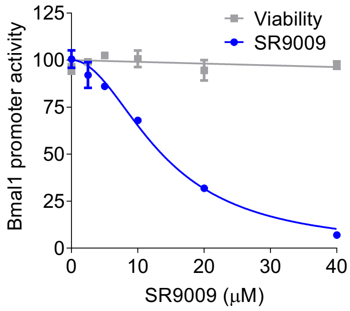

Supplementary Fig.2: REV-ERB agonist SR9009 inhibits Bmal1 promoter activity without cytotoxicity. HepaRG cells stably expressing Bmal1-luciferase promoter were treated with SR9009 for 48h and luciferase activity measured. Cytotoxicity was determined using an LDH assay (mean ± S.E.M., n = 3). Source data are provided as a Source Data file.

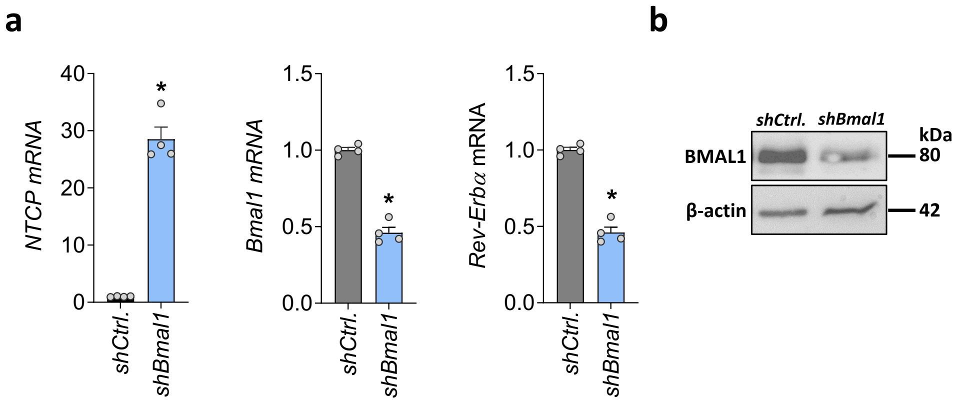

Supplementary Fig.3: Silencing BMAL1 increases NTCP transcripts.

(a) Total RNA was extracted from control or *Bmal1* silenced HepaRG cells and *NTCP, Bmal1* and *Rev-Erbα* mRNA levels measured by qRT-PCR. Data are expressed relative to control (mean ± S.E.M., n = 4, Mann–Whitney test, Two-sided). **(b)** Bmal1 silencing was validated by western blotting. ∗ denotes p < 0.05. Source data are provided as a Source Data file.

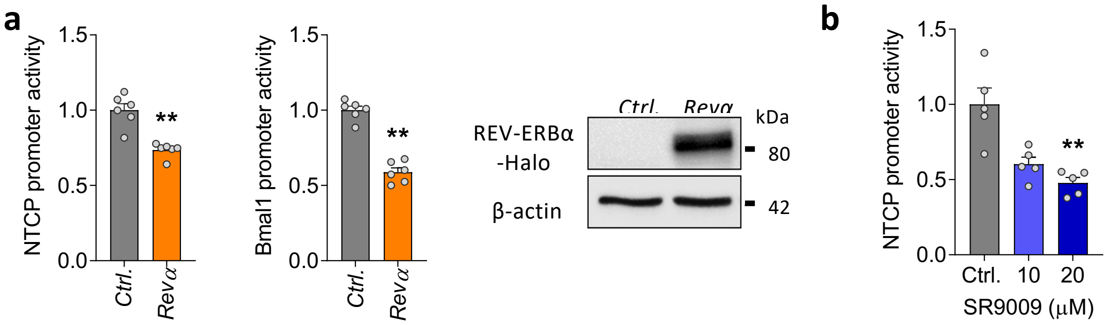

Supplementary Fig.4: REV-ERBα as a repressor of NTCP promoter activity.
(a) Huh-7.5 hepatoma cells were co-transfected with NTCP or Bmal1 promoter luciferase reporters with control or *Rev-Erbα* (Halo-tagged) expression plasmid. Promoter activities were determined 48h later by quantifying luciferase activity. Data are expressed relative to control (mean ± S.E.M., n = 6, Mann–Whitney test, Two-sided). REV-ERBα overexpression was validated by western blotting using an anti-Halo antibody. **(b)** Huh-7.5 hepatoma cells were transfected with NTCP promoter luciferase reporter for 48h and treated with SR9009 for 24h and promoter activity determined by quantifying luciferase activity. Data are expressed relative to control treatment (mean ± S.E.M., n = 5, Kruskal–Wallis ANOVA with Dunn's test). ∗ denotes $p < 0.05$, ∗∗ < 0.01. Source data are provided as a Source Data file.

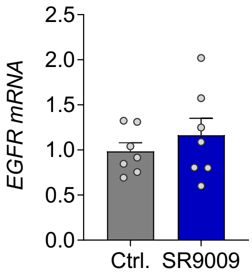

Supplementary Fig.5: REV-ERB agonist SR9009 does not regulate EGFR mRNA.
Differentiated HepaRG cells were treated with SR9009 (20 μM) for 24h and *EGFR* RNA measured by qRT-PCR (mean ± S.E.M., n = 7, Mann–Whitney test). Source data are provided as a Source Data file.

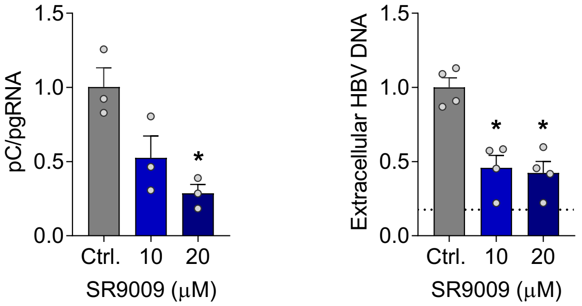

Supplementary Fig.6: REV-ERB agonist SR9009 treatment reduces HBV pC/pgRNA levels and extracellular HBV DNA.
HepG2.215 cells were treated with SR9009 for 48h and pC/gRNA levels determined by qRT-PCR. Data are expressed relative to untreated (Ctrl) cells (Mean ± S.E.M., n = 3, Kruskal–Wallis ANOVA with Dunn's test). Extracellular HBV DNA levels were determined by qPCR. The reverse transcriptase inhibitor Entecavir (ETV, 1 µM) was included as positive control. Data are expressed relative to untreated (Ctrl) cells (Mean ± S.E.M., n = 4, Kruskal–Wallis ANOVA with Dunn's test). ∗ denotes $p < 0.05$. Source data are provided as a Source Data file.

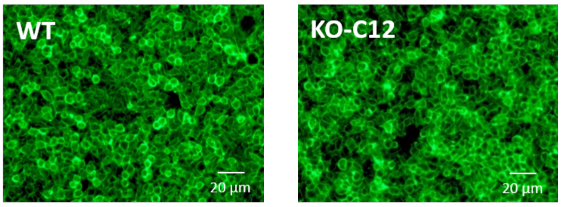

Supplementary Fig.7: NTCP expression in wild type and Rev-Erbα knockout HepG2 cells.
Parental (WT) or *Rev-Erbα* KO (C12) HepG2-NTCP cells were stained with Alexa 488 fluorophore labelled Myrcludex B (200 nM) and images acquired using a Zeiss fluorescence microscopy. Representative fluorescent images were shown (n = 3 for each cell line)

E-box	Position	Sequence (CANNTG)	Genome location
1	597-602	CACCTG	S/ POLYMERASE (RT)
2	689-694	CATTTG	S / POLYMERASE (RT)
3	1179-1184	CAAGTG	POLYMERASE (RNASE H)
4	1397-1402	CAACTG	POLYMERASE (RNASE H) / X
5	1566-1571	CATCTG	POLYMERASE (RNASE H) / X
6	2306-2311	CAAATG	CORE / POLYMERASE (TP)
7	2630-2635	CAATTG	POLYMERASE (TP)
8	2901-2906	CAGTTG	POLYMERASE (SPACER) / PRE S
9	2966-2971	CACCTG	POLYMERASE (SPACER) / PRE S

RORE	Position	Sequence (RGGTCA)	Genome location
1	2816-2821	GGGTCA	TP

Supplementary Fig.8: Circadian regulatory E-box and RORE motifs in HBV genome (Genotype D, ayw).

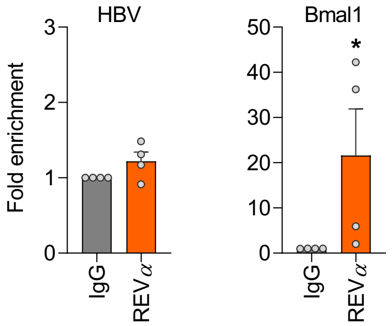

Supplementary Fig.9: ChIP-qPCR evaluation of REV-ERBα binding HBV DNA.
Chromatin extracts from HepG2-pEpi cells were immunoprecipitated using antibodies specific for REV-ERBα or rabbit IgG as a negative control. PCR for HBV DNA using primers targeting the RORE or control host gene Bmal1 was performed and %IP data presented relative to the rabbit IgG control shown as the dotted line (mean ± S.E.M., n = 4, Mann–Whitney test, Two-sided). ∗ denotes $p < 0.05$. Source data are provided as a Source Data file.

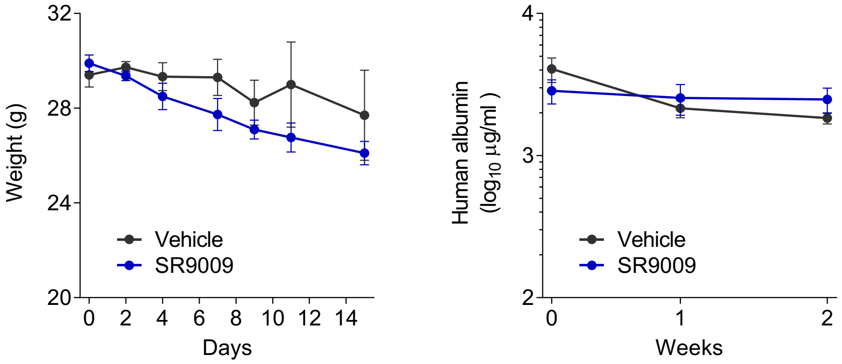

Supplementary Fig. 10: Effect of REV-ERB agonist SR9009 on HBV infected mice weight and albumin expression. FRG-NOD mice transplanted with primary human hepatocytes were infected with 10^9 HBV genome equivalents and 8 weeks later the mice were treated twice a day by intraperitoneal injection with either vehicle or SR9009 (100 mg/kg) for 2 weeks. Individual animal weights were recorded and peripheral human albumin quantified weekly (mean ± S.E.M., n = 3). Source data are provided as a Source Data file.

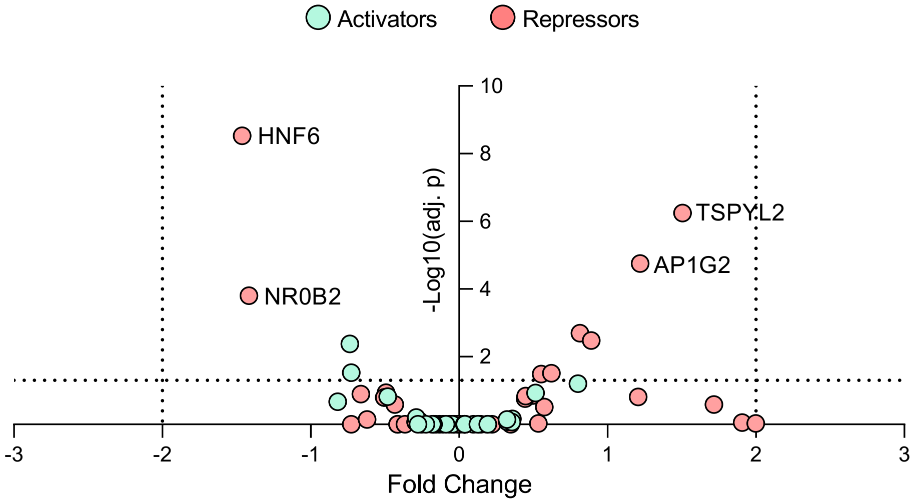

Supplementary Fig.11: Transcriptomic analysis of REV-ERB agonist SR9009 and vehicle treated chimeric mice.

Analysis of known activators and repressors of HBV was performed and fold change were plotted against the FDR. Gene names with p value < 0.001 were shown. Fold Change of +/- 2 is deemed statistically significant. Source data are provided as a Source Data file.

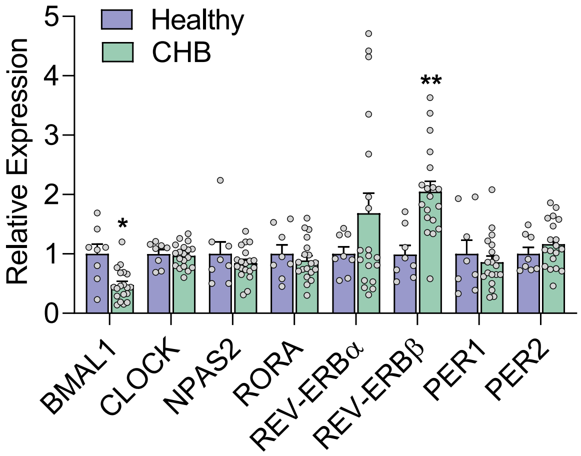

Supplementary Fig.12: Core clock gene transcripts in chronic hepatitis B and healthy subjects.

Expression of core clock genes in liver biopsy tissue from healthy (n=8) or hepatitis B virus infected patients classified according to EASL 2017 guidelines in the HBeAg negative phase of disease with chronic hepatitis (n = 19) or chronic infection (n = 8). Data are presented relative to healthy controls, statistics were tested with multiple Mann-Whitney U tests, Two-sided, and p values adjusted with Bonferroni correction ($P<0.05$ deemed statistically significant). ∗ denotes $p < 0.05$, ∗∗ < 0.01. Source data are provided as a Source Data file.

Supplementary Table 1: List of primers used

Taqman expression assays	Supplier	Cat #.
ARNTL (Bmal1) TaqMan® Gene Expression Assay	ThermoFisher	Hs00154147
NR1D1 (Rev-Erbα) TaqMan® Gene Expression Assay	ThermoFisher	Hs00253876
SLC10A1 (NTCP) TaqMan® Gene Expression Assay	ThermoFisher	Hs00161820
SCD TaqMan® Gene Expression Assay	ThermoFisher	Hs01682761
FASN TaqMan® Gene Expression Assay	ThermoFisher	Hs01005622
HBV-pgRNA TaqMan® Gene Expression Assay	ThermoFisher	AIKAMSS
b2M Control Mix	Applied Biosystems	4325797
**qPCR Primers (5'-3')**		
HBV internalization assay DNA forward: ACTCACCAACCTCCTGTCCT		
HBV internalization assay DNA reverse: GACAAACGGGCAACATACCT		
HBV internalization assay DNA probe: (FAM)-TATCGCTGGATGTGTCTGCGGCGT-(TAMRA)		
PrP forward: TGCTGGGAAGTGCCATGAG		
PrP reverse: CGGTGCATGTTTTCACGATAGTA		
rcDNA forward: GTTGCCCGTTTGTCCTCTAATTC		
rcDNA reverse: GGAGGGATACATAGAGGTTCCTTGA		
cccDNA forward: GCCTATTGATTGGAAAGTATGT		
cccDNA reverse: AGCTGAGGCGGTATCTA		
b2M forward: CTACACTGAATTCACCCCCACTG		
b2M reverse: ACCTCCATGATGCTGCTTACATG		
b-Actin forward: CCAACCGCGAGAAGATGA		
b-Actin reverse: CCAGAGGCGTACAGGGATAG		
EGFR forward: AACACCCTGGTCTGGAAGTACG		
EGFR reverse: TCGTTGGACAGCCTTCAAGACC		
**ChIP-PCR Primers (5'-3')**		
Per1 promoter forward: GTCAAGGAAAATCCCCAGCTTCTG		
Per1 promoter reverse: CCAAGATTGGTGACGTAAATGCCA		
Bmal1 promoter forward: TTGGGCACAGCGATTGGT		
Bmal1 promoter reverse: GTAAACAGGCACCTCCGTCC		
NTCP-Ebox1 forward: AAATCTGTCCCCACCTCCACCC		
NTCP-Ebox1 reverse: TGGTTGGAACCTAGGAGGCCTG		
NTCP-Ebox2 forward: ACCCAGGCCTCCTAGGTTCCAA		
NTCP-Ebox2 reverse: GGGCCACCTAGTGTGTGAGGTT		
NTCP-Ebox3 forward: CACAGCCAGGGTTCTCTCAGGT		
NTCP-Ebox3 reverse: AATACTGGTGGTGGCTGCTCCA		
NTCP-Ebox4 forward: AACCAAAGGCAACACCCAGCTC		
NTCP-Ebox4 reverse: TCCTTTTCCCAGCTCCGCTCTT		
NTCP-Ebox5 forward: AGGAGCTGCAGGGAAGGAGATG		
NTCP-Ebox5 reverse: TGTCACTTTGCTGCTTCTGGGC		
NTCP-RORE1 forward: GCATCCTCTCAGCTCCCAGGAA		
NTCP-RORE1 reverse: AGCTGGGTGTTGCCTTTGGTTC		
NTCP-RORE2 forward: GCCCAGAAGCAGCAAAGTGACA		
NTCP-RORE2 reverse: CCCCAAAGAGGAACAGCCCAGA		
HBV-RORE forward: TTACACACTCTATGGAAGGCGG		
HBV-RORE reverse: AAAGATTCTGCCCCATGCTGTA		
HBV-E-box2 forward (680-703): ACTAGTGCCATTTGTTCAGTGGT		
HBV-E-box2 reverse (804-827): ACCCAAAGACAAAAGAAAATTGGT		
HBV-E-box3 forward (1168-1188): CAGGTCTGTGCCAAGTGTTTG		
HBV-E-box3 reverse (1278-1299): CAAAACAAGCGGCTAGGAGTTC		
HBV-E-box4 forward (1305-1328): AGCAGGTCTGGAGCAAACATTATC		
HBV-E-box4 reverse (1413-1434): GGACGTAAACAAAGGACGTCCC		
HBV-E-box5 forward (1153-1573): GTCTGTGCCTTCTCATCTGCC		
HBV-E-box5 reverse (1630-1652): AGACCTTGGGCAATATTTGGTGG		
HBV-E-box6 forward (2294-2317): CTTATAGACCACCAAATGCCCCTA		
HBV-E-box6 reverse (2396-2414): GGCGATTGAGACCTTCGTC		
