# Supplementary material for: Circadian control of hepatitis B virus replication
Source: Nat Commun. 2021 Mar 12;12:1658. doi: 10.1038/s41467-021-21821-0 (PMC7955118; doi:10.1038/s41467-021-21821-0)
Supplement: Supplementary file 3 — Reporting Summary [file 41467_2021_21821_MOESM3_ESM.pdf]

nature research

Corresponding author(s): Jane A McKeating
Xiaodong Zhuang

Last updated by author(s): Jan 11, 2021

Reporting Summary

Nature Research wishes to improve the reproducibility of the work that we publish. This form provides structure for consistency and transparency in reporting. For further information on Nature Research policies, see our Editorial Policies and the Editorial Policy Checklist.

Statistics

For all statistical analyses, confirm that the following items are present in the figure legend, table legend, main text, or Methods section.

n/a	Confirmed	
☐	☒	The exact sample size (*n*) for each experimental group/condition, given as a discrete number and unit of measurement
☐	☒	A statement on whether measurements were taken from distinct samples or whether the same sample was measured repeatedly
☐	☒	The statistical test(s) used AND whether they are one- or two-sided<br>*Only common tests should be described solely by name; describe more complex techniques in the Methods section.*
☒	☐	A description of all covariates tested
☒	☐	A description of any assumptions or corrections, such as tests of normality and adjustment for multiple comparisons
☐	☒	A full description of the statistical parameters including central tendency (e.g. means) or other basic estimates (e.g. regression coefficient) AND variation (e.g. standard deviation) or associated estimates of uncertainty (e.g. confidence intervals)
☐	☒	For null hypothesis testing, the test statistic (e.g. *F*, *t*, *r*) with confidence intervals, effect sizes, degrees of freedom and *P* value noted<br>*Give P values as exact values whenever suitable.*
☒	☐	For Bayesian analysis, information on the choice of priors and Markov chain Monte Carlo settings
☒	☐	For hierarchical and complex designs, identification of the appropriate level for tests and full reporting of outcomes
☒	☐	Estimates of effect sizes (e.g. Cohen's *d*, Pearson's *r*), indicating how they were calculated

Our web collection on statistics for biologists contains articles on many of the points above.

Software and code

Policy information about availability of computer code

Data collection	No software was used for data collection
Data analysis	All statistical analyses were performed using GraphPad Prism 7 and 8;<br>All cartoons were created with BioRender.com;<br>For RNA-seq analysis, sequence adapters were removed and reads trimmed by Trim Galore v0.5.079. The reads were mapped against the reference mouse genome (mm10/GRCm38) and reference human genome (hg38/GRCm38) using STAR v2.5.3 80. Counts per gene were calculated using Rsubread v1.28.1 81. Reads were analyzed by edgeR v3.30.082, normalized using TMM. Mouse genes were converted to human orthologs using biomaRt v2.44.0 83and the built-in ensembl datasets for human and mouse. Pathway analysis using the Human KEGG 2019 library was performed using enrichR v2.184;<br>The publicly available gene expression microarray dataset (GSE34018) was obtained using GEOquery v2.56.0 and mapped to genes using illuminaMousev2.db v1.26.0. Differential expression analysis, controlling for time of day, was performed using edgeR v3.30.0. Genes were converted to their human orthologs using biomaRt v2.44.0 and the ensembl datasets used to compare human and mouse transcriptomes.

For manuscripts utilizing custom algorithms or software that are central to the research but not yet described in published literature, software must be made available to editors and reviewers. We strongly encourage code deposition in a community repository (e.g. GitHub). See the Nature Research guidelines for submitting code & software for further information.

Data

Policy information about availability of data

All manuscripts must include a data availability statement. This statement should provide the following information, where applicable:

- Accession codes, unique identifiers, or web links for publicly available datasets
- A list of figures that have associated raw data
- A description of any restrictions on data availability

The publicly available gene expression microarray dataset (GSE3401852) was used; The RNA-seq data from SR9009 treated mice have been deposited at NCBI GEO with the identifier GSE163285 and can be accessed via: https://www.ncbi.nlm.nih.gov/geo/query/acc.cgi?acc=GSE163285. The authors declare that all data supporting the findings of this study are available within the Source data file.

Field-specific reporting

Please select the one below that is the best fit for your research. If you are not sure, read the appropriate sections before making your selection.

☒ Life sciences ☐ Behavioural & social sciences ☐ Ecological, evolutionary & environmental sciences

For a reference copy of the document with all sections, see nature.com/documents/nr-reporting-summary-flat.pdf

Life sciences study design

All studies must disclose on these points even when the disclosure is negative.

Sample size	For most experiments this was achieved using triplicate measurements. Where used, higher replicate numbers are indicated in the text.
Data exclusions	No data were excluded
Replication	Individual experiments were reproduced three times in an independent manner with similar results.
Randomization	Not relevant for our study: only one variable is tested in each experiment
Blinding	Not relevant for our study: only one variable is tested in each experiment

Reporting for specific materials, systems and methods

We require information from authors about some types of materials, experimental systems and methods used in many studies. Here, indicate whether each material, system or method listed is relevant to your study. If you are not sure if a list item applies to your research, read the appropriate section before selecting a response.

Materials & experimental systems

n/a	Involved in the study	
☐	☒	Antibodies
☐	☒	Eukaryotic cell lines
☒	☐	Palaeontology and archaeology
☐	☒	Animals and other organisms
☒	☐	Human research participants
☒	☐	Clinical data
☒	☐	Dual use research of concern

Methods

n/a	Involved in the study	
☒	☐	ChIP-seq
☒	☐	Flow cytometry
☒	☐	MRI-based neuroimaging

Antibodies

Antibodies used	The following primary antibodies were used: anti-BMAL1 (1 µg/mL, Ab93806, Abcam, UK), anti-BMAL1 (1 µg/mL, ChIP Grade, Ab3350), anti-NTCP (HPA042727, ATLAS), anti-HDAg (Scrum), anti-Halotag (1 mg/mL, G9211, Promega, UK), anti-REV-ERBα (1 µg/mL, PA5-29865, Thermo Fisher Scientific, UK), anti-HNF4A (1 µg/mL, ChIP Grade, Ab181604, Abcam, UK), anti-β-actin (A5441, Sigma, UK) and Rabbit IgG (NI01, Sigma, UK).
Validation	All commercially available antibodies were validated by their respective manufacturer, listed below:<br>anti-BMAL1: https://www.abcam.com/bmal1-antibody-ab93806.html<br>anti-BMAL1 (ChIP Grade): https://www.abcam.com/bmal1-antibody-ab3350.html<br>anti-NTCP: https://www.sigmaaldrich.com/catalog/product/sigma/hpa042727?lang=en&region=GB<br>anti-Halotag: https://www.promega.co.uk/products/protein-detection/primary-and-secondary-antibodies/anti-halotag-monoclonal-antibody/?catNum=G9211<br>anti-REV-ERBα: https://www.thermofisher.com/antibody/product/NR1D1-Antibody-Polyclonal/PA5-29865

anti-HNF4A: https://www.abcam.com/hnf-4-alpha-antibody-epr16885-chip-grade-ab181604.html
anti-β-actin: https://www.sigmaaldrich.com/catalog/product/sigma/a5441?lang=en&region=GB
Rabbit IgG: https://www.sigmaaldrich.com/catalog/product/mm/ni01?lang=en&region=GB

Eukaryotic cell lines

Policy information about cell lines

Cell line source(s)	The human hepatoma cell line HepG2-NTCP were provided by Stefan Urban (University of Heidelberg). HepG2.2.15, HepG2-pEpi and HepaRG cells were provided by Ulrike Protzer, Technische Universität München, Munich.
Authentication	None of the cell lines were authenticated
Mycoplasma contamination	Mycoplasma testing was routinely performed on cell lines and verified as negative.
Commonly misidentified lines (See ICLAC register)	None

Animals and other organisms

Policy information about studies involving animals; ARRIVE guidelines recommended for reporting animal research

Laboratory animals	Six-week-old FRG-NOD mice were housed and bred at the INSERM U1110 animal facility (regional agreement n° E-67-482-7), and were kept under 2-(2-nitro-4-trifluoromethylbenzoyl)-1,3-cyclohexanedione (NTBC) in drinking water at 16 mg/L.
Wild animals	No wild animal enrolled in this study.
Field-collected samples	This study did not involve samples collected from the field.
Ethics oversight	The transplantation procedure was approved by the local Ethics committee and authorized by the French ministry of research and higher education (APAFIS#4485-2016031115352125 v3).The experimental procedure was approved by the local Ethics committee and authorized by the French ministry of research and higher education (APAFIS#13872-2018050214497349 v1).

Note that full information on the approval of the study protocol must also be provided in the manuscript.
